# Communication about distress and well-being: Epistemic and ethical considerations

**DOI:** 10.1177/13634615221082795

**Published:** 2022-03-18

**Authors:** Ross G. White, Richard Fay, Anna Chiumento, Catalina Giurgi-Oncu, Alison Phipps

**Affiliations:** 1 School of Psychology, Queen’s University Belfast, Belfast, UK; 2 Manchester Institute of Education, School of Environment, Education and Development, The University of Manchester, Manchester, UK; 3 Institute of Population Health, University of Liverpool, Liverpool, UK; 4 162271 Universitatea de Medicina si Farmacie, Victor Babes din Timisoara, Neuroscience Department, Timisoara, Romania; 5 School of Education, University of Glasgow, Glasgow, UK

**Keywords:** distress, epistemic injustice, epistemology, global mental health, interpretation, multi-language, reflective practice, reflexivity, translation, well-being

## Abstract

Communication about well-being and distress involves multiple stakeholders, including experts by experience (EBE), researchers, clinical practitioners, interpreters, and translators. Communication can involve a variety of discourses and languages and each of the stakeholders may employ diverging epistemologies to understand and explain experiences. These epistemologies may link to different sources of authority and be articulated using particular linguistic resources. *Epistemic injustice* can occur when stakeholders, intentionally or unintentionally, fail to recognise the validity of other stakeholders’ ways of conceptualising and verbalising their experience of well-being and distress. Language lies at the heart of the risk of epistemic injustice involved in the process of expressing well-being and distress as seen in: 1) the interface between divergent discourses on well-being and distress (e.g., biomedical vs. spiritual); and 2) communications involving multiple linguistic resources, which can be subdivided into multi-language communications involving a) translation of assessment measures, and b) interpreted interactions. Some of the challenges of multi-language communication can be addressed by translators or interpreters who strive for conceptual equivalence. We argue, however, that all stakeholders have an important role as “epistemic brokers” in the languaging of possible epistemological differences. Effective epistemic brokering requires that all stakeholders are reflexively and critically aware of the risks of epistemic injustice inherent in multi-language communication. The article concludes with a set of prompts to help raise stakeholder awareness and reflexivity when engaging in communication about well-being and distress.

## Introduction

Clinical and research communication about well-being and distress involves multiple stakeholders, including experts by experience (EBE), researchers, clinical practitioners, interpreters, and translators. Communication often also involves multiple discourses and languages. Each stakeholder may understand and explain experiences using potentially divergent epistemologies, linked to differing sources of authority, and articulated using particular linguistic resources. Drawing on Christison and Murray's (2020, p. 1) definition of *languaging* as a “process of making meaning and shaping knowledge and experiences through language”, we term this process of communication the “languaging of well-being and distress”. If stakeholders, intentionally or unintentionally, fail to recognise the validity of other stakeholders’ ways of conceptualising and verbalising their experience of well-being and distress, this can result in *epistemic injustice* which has been defined as “a wrong done to someone specifically in their capacity as a knower” (Fricker, 2007, p. 1). Although terms such as *idioms* or *cultural concepts of distress* have been introduced to recognise diverse ways of understanding well-being and distress, in seeking to value particular ethno-psychologies, they may inadvertently reinforce the proposed universality of non-localised, diagnostic terms that clinicians and/or researchers may continue to reference. Thus, the risk of epistemic injustice remains.

Language lies at the heart of the risks of epistemic injustice involved in the languaging of well-being and distress. Its problematic presence can be seen in: 1) the interface between divergent discourses on well-being and distress (e.g., biomedical vs. spiritual) and 2) communications involving multiple linguistic resources. The latter type can be subdivided into multi-language communications involving a) translation of assessment measures and b) interpreted interactions. While some of the multi-language challenges of communication can be addressed by translators and/or interpreters as, for example, they strive for conceptual equivalence, we argue that *all* stakeholders have an important role as *epistemic brokers* (Raymond, 2014) in the languaging of possible epistemological differences. More equitable communication requires effective epistemic brokering. In turn, effective epistemic brokering requires stakeholders to be reflexively and critically aware of the risks of epistemic injustice inherent in health communication.

In this article, we discuss Fricker’s (2007) concept of epistemic injustice in the context of existing literature on the languaging of well-being and distress. We then consider examples of ways that mental health practitioners and researchers have sought to engage in epistemic brokering in multi-language communications, such as by incorporating local idioms of distress, and adopting approaches to translating communication about well-being and distress across languages. To address the need for greater epistemic pluralism, we present a set of prompts designed to encourage all stakeholders involved in the communication to be critically aware and reflexive about the possible differences, and origins of these differences, in epistemology and power. We hope that application of these prompts will minimise the risk of potential harm that can arise from epistemic divergences in communication—particularly multi-language communication—about well-being and distress. Examples from both clinical practice and research studies will be used to illustrate key points. We recognise that these different contexts pose unique, as well as overlapping, challenges and opportunities.

## Epistemic injustice and the languaging of well-being and distress

In her consideration of wrongs perpetrated against someone specifically in their capacity as a knower, Fricker (2007) identifies two forms of *epistemic injustice*. First, *testimonial injustice*, which occurs “when prejudice causes a hearer to give a deflated level of credibility to a speaker's word” (p. 1), for example, when “the police do not believe you because you are black”—a form of injustice “caused by prejudice in the economy of credibility” (p. 1). Second, *hermeneutical injustice*, which occurs “when a gap in collective interpretive resources puts someone at an unfair disadvantage when it comes to making sense of their social experiences”, for example, when “you suffer sexual harassment in a culture that still lacks that critical concept”, a form of injustice “caused by structural prejudice in the economy of collective hermeneutical resources” (p. 1).

There is a growing body of literature examining epistemic injustice in the languaging of well-being and distress. Kidd and Carel (2017) explore epistemic injustice in the context of healthcare settings, specifically with regard to consultations, medical education and policymaking. Blease et al. (2017) suggest that epistemic injustice can lead to “patient harm” (e.g., marginalisation of service users in their care and treatment). In healthcare settings, testimonial injustices such as the stereotyping of individuals and the associated downgrading of the credibility of their accounts, such as tokenistic involvement of EBE in designing research studies, can arise (Blease et al., 2017). There can also be hermeneutical injustices in healthcare settings related to the purported causes of potential conditions. This can lead to a lack of access to shared conceptual frameworks between healthcare professionals and EBE for facilitating a common understanding of well-being and distress. Watters (2017) suggests that mental health literacy campaigns seeking to increase people's awareness of mental disorders (as classified in diagnostic manuals used by the World Health Organization [WHO] and the American Psychiatric Association [APA]) might risk perpetrating what he termed *epistemic violence*^1^ and the potential subjugation of indigenous ways of understanding well-being and distress. Similarly, de Sousa Santos’s (2015) term *epistemicide* captures the detrimental impact that the dominant Western epistemology—or “monoculture of scientific knowledge” (de Sousa Santos, 2015, p. 207) as he terms it—might have on epistemologies in the Global South.

## Intersubjectivity and epistemic injustice

As with all communication, interactions about well-being and distress are *inter*subjective, i.e., they involve the interplay of the differing perceptions of reality held by stakeholders involved in the communication (Munroe, 2011; Zhao & Zhu, 2013). This *intersubjectivity*, as facilitated through language, brings different ways of knowing into play, thereby raising the possibility of epistemic injustice. For example, as one stakeholder (e.g., EBE, clinician, or researcher) in the communication interacts with another, their prejudices regarding the credibility of other stakeholders may lead them to devalue what that individual communicates, and in turn shape how they themselves will communicate. This may happen because of how they see the other as a type of person (i.e., testimonial injustice); or might also result from the limited overlap in the conceptual frameworks to which the stakeholders have access (i.e., hermeneutical injustice).

**Figure 1.**
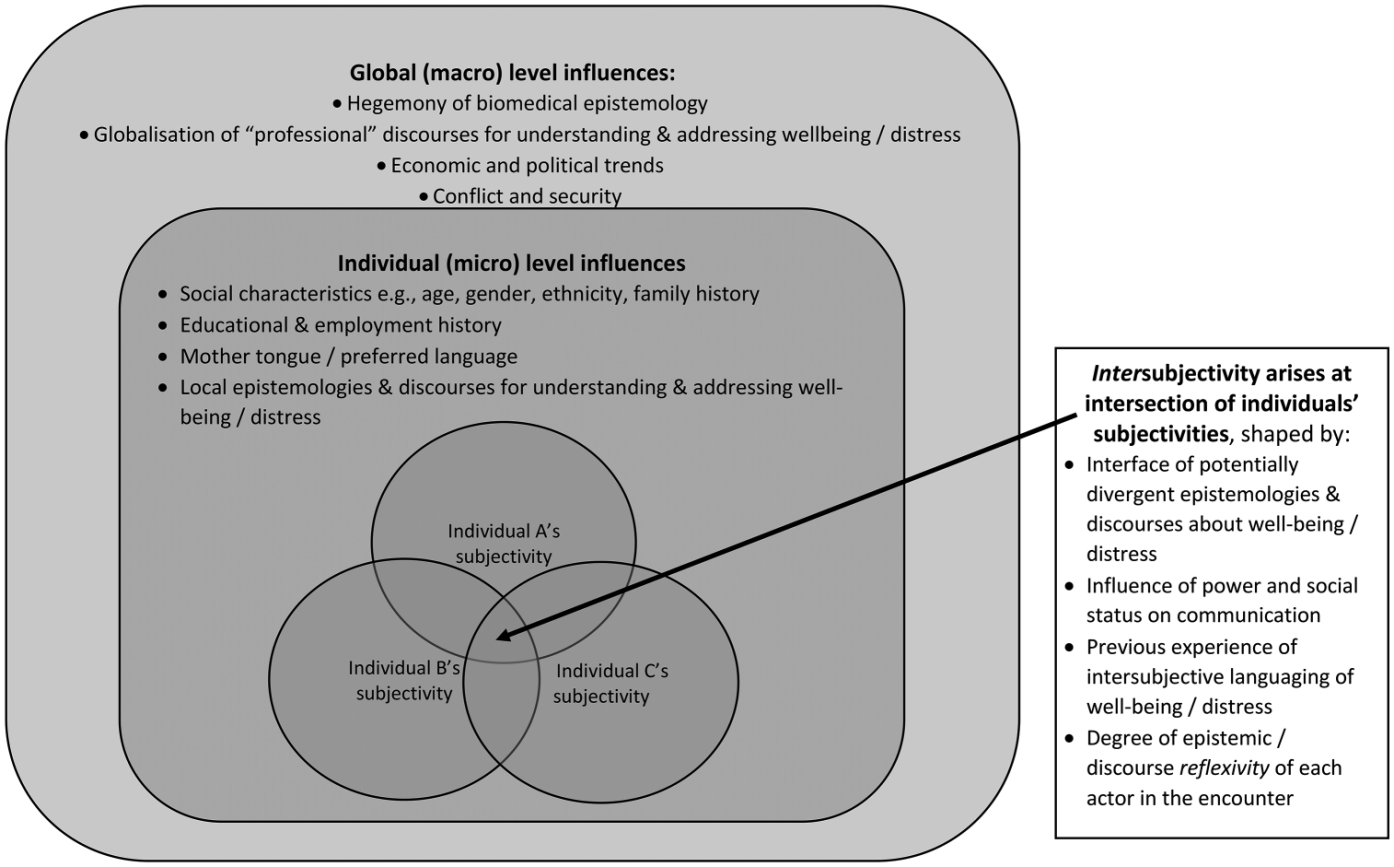
Factors contributing to intersubjectivity in communication about well-being and distress.

Drawing upon this body of literature, Figure 1 provides a diagrammatic representation of important elements in intersubjectivity and associated risks of epistemic injustice in communication about well-being and distress. These elements operate at the global/macro level and the individual/micro-level. Both levels may be shaping the languaging of well-being and distress. If communication is to avoid the risk of epistemic injustice, both levels need to be brokered.

## Divergences in discourses about distress and well-being

Across time, space, languages, and cultures, different understandings of, and verbalisations about, emotional and psychological distress (herein referred to collectively as “distress”) have developed. In the West, contemporary communication about distress has been heavily influenced by diagnostic labels often originally verbalised through English. The influence of agencies and networks, such as the *World Health Organization* and *World Psychiatric Association*, means that the nomenclature of “mental disorders” has been used to describe, categorise, and diagnose based on criteria that are published in manuals such as the *Diagnostic and Statistical Manual of Mental Disorders* (5th ed.; *DSM-5*; American Psychiatric Association [APA], 2013a) and the *International Classification of Diseases* (ICD-11; World Health Organization, 2019). The validity and reliability of psychiatric diagnostic criteria continue to be keenly debated, with concern regarding the appropriateness of the ways that distress is conceptualised and described; and the systems and treatments that it legitimises (Allsopp et al., 2019). This is not simply a philosophical matter: discordances in terminology between a health professional and an EBE can discourage an EBE from participating in consultations (Derose & Baker, 2000; Rivadeneyra et al., 2000; Roter & Hall, 2006).

One response to such concerns is the recognition that distress can be understood and communicated in ways particular to local contexts. Thus, linguistic and cultural variation in the experience of distress has been recognised through concepts such as *cultural concepts of distress* (APA, 2013a) and *idioms of distress* (Nichter, 1981), the latter defined as “socially and culturally resonant means of experiencing and expressing distress in local worlds” (Nichter, 2010, p. 405). There is also a growing interest in local, culturally salient ways of communicating the capacity to overcome difficulties - what can be termed *idioms of resilience* (Kim et al., 2019). Calls to increase research activity focusing on cultural concepts of distress have been made, including undertaking epidemiological and anthropological investigations (Kohrt et al., 2014). It has been suggested that practitioners in the West can be subject to *idiomatic blindspots* (Cork et al., 2019) when they fail to recognise local distinctions in how experiences of distress are understood and communicated. We argue that there is a need for greater *epistemic pluralism*, and a greater awareness, globally, of the risk of epistemic injustices in communication about experiences of distress and well-being. The prompts with which we end this article are a first step to support the development of such awareness by all the stakeholders involved.

Although *idioms of distress* serve important communicative and empathetic functions for people who share common cultural beliefs and practice, they can pose challenges for communication about distress with people who espouse different cultural beliefs and practices. Keys et al. (2012) illustrated the pitfalls of these intercultural exchanges in their research in post-earthquake Haiti. A total of 17 idioms of distress were identified, pertaining to emotional, cognitive, and psychosocial distress. Over half of these related to *tèt* (head, e.g., tèt virè; turned head) or *kè* (heart, e.g., kè serè; tight/bound heart) in Haitian *ethno-psychology*, the system by which the self, emotions, human nature, motivation, personality, and the interpretation of experience are conceptualised by members of the cultural group (Kirmayer, 1989; White, 1992). Keys et al. (2012) include a vignette to illustrate the markedly different support that a distressed Haitian person may have been offered from an international aid worker who was knowledgeable about the idioms, as compared to support offered by someone who was not. For Keys et al. (2012), a lack of attention to idioms of distress may lead to missed opportunities to provide psychosocial support and culturally appropriate forms of advice and guidance.

Most recently, the term *cultural concepts of distress* (CCD) has been coined to capture “ways that cultural groups experience, understand, and communicate suffering, behavioral problems, or troubling thoughts and emotions” (APA, 2013a; p. 787). Nine examples of *cultural concepts of distress* are listed in a glossary in DSM-5 (APA, 2013a), including: *khyal cap* (Khmer: “wind attacks”) and *taijin kyofusho* (Japanese: “interpersonal fear disorder”). It is striking that none of these nine illustrative examples of CCDs were English-language terms. The erroneous impression this potentially creates is that manifestations and expressions of distress amongst those who speak English as a first or main language are less likely to be unique to variations in cultural beliefs and practice. In addition, to gather culturally relevant information in clinical assessments, the *Cultural Formulation Interview* (CFI) was added to the 5th edition of the DSM (APA, 2013b).

Although the concepts listed in the DSM-5 glossary can be translated (as they have been into English in the paragraph above), or interpreted into other languages, it is not clear how well the phenomenology of a person's direct conscious experience might apply across different contexts. The processes of textual translation or oral interpretation risk detaching CCDs from their contextual moorings and may direct the reader/listener towards epistemic frames that are distinct from those in the context where the cultural concepts of distress emerged. Lewis-Fernández and Kirmayer (2019) provide a comprehensive account of how work relating to CCDs has evolved across time, the contribution that social science research has made to this, and what future directions this work can take.

## Multi-language communication about distress and well-being

A significant proportion of the global population has access to linguistic resources in more than one language. As such, communications can involve aspects of various languages rather than being restricted to the mother-tongue language ascribed to the individuals involved. We argue that the term *multi-language communication* accommodates the possibility that various linguistic resources may be used flexibly by different stakeholders—all of whom will have varying levels of expertise in the languages that might be in play. We propose that stakeholders engaging in multi-language communication should remain open to the possibility of being complicit in epistemic injustices. Meaningful and accurate interpretation and translation of distress-related concepts will not be sufficient to prevent epistemic injustice, as this may not capture the intersubjectivities inherent to communication and the associated risk of what Raymond (2014) referred to as a “steep epistemic gradient” (i.e., a marked disparity in the epistemologies used by the stakeholders involved). In light of this context, it is important to critically explore the ways epistemic brokering can be managed. Here, we focus on two specific forms of multi-language communication relating to mental health in which epistemic injustice might arise: a) the translation of assessment scales developed in the West; and b) interpreted communications about experiences of well-being and distress.

## Translating assessment scales

To facilitate international comparisons of levels of well-being and distress, researchers commonly translate and/or culturally adapt assessment instruments for use in different cultural and linguistic contexts. This can, for example, facilitate transnational collection of data that allows international agencies to chart global trends in mental health epidemiology, and allows researchers and clinicians to make comparisons between work being undertaken in different contexts. The *Centre for Epidemiologic Studies Depression Scale* (CES-D; Radloff, 1977), originally developed in the US, is an example of one such assessment instrument that was translated and culturally adapted for use in Northern Uganda. Singla et al. (2015) and Natamba et al. (2014) both report on their use of the *Luo* language version of the CES-D (“Luo” being the term that collectively refers to the *Langi* and *Acholi* languages widely spoken in Northern Uganda^2^). The methodological description of how the issue of language is addressed in the Natamba et al. (2014) paper is fairly typical of how the instrument translation and adaptation process is approached in global mental health research: “All study instruments were translated by local research staff into Acholi and Langi, the two predominant and closely related Luo languages that are spoken in the study communities. The questionnaires were then back-translated into English by the same team, and discrepancies in conceptual and semantic equivalence were “resolved” through discussion involving all the translators, the research assistants, and the psychiatrist” (Natamba et al., 2014, p. 2).

The CES-D includes items such as: “I felt that I could not shake off the blues even with help from my family” (item 3) and “I felt depressed” (item 6). These items embed English language idioms, “shake off the blues”, and diagnostic terms, “depressed”, both of which make translation complex. Neither the Singla et al. (2015) nor the Natamba et al. (2014) papers provide further details about the wording of the Luo version of the CES-D, or the potential limitations these translations may have posed when conducting mental health assessments. The key tension here is the extent to which a measure developed and validated in one cultural context should, and can, be *sufficiently* adapted to reflect how distress is experienced and communicated in another. The original English language wording of the CES-D items reflects a particular ethno-psychological perspective, and there may be variation in the generalisability of these items both within and between speakers of different languages. One element frequently considered in the translation and cultural adaptation of assessment instruments is *equivalence* across languages.

## Translation and the concept of “equivalence”

Natamba et al. (2014) mention the concepts of *conceptual* and *semantic equivalence* which are frequently applied when translating health-related research. Flaherty et al. (1988, p. 258) propose five forms of equivalence in translation: (1) *content equivalence*, i.e., each item of an assessment measure is relevant to the phenomenon of the cultures being investigated; (2) *semantic equivalence*, i.e., each item has the same meaning in a particular cultural frame following translation into the languages/idioms of other cultures; (3) *technical equivalence*, i.e., the mode of assessment (e.g., interview, written self-report etc.) is comparable with regard to the data that it produces in the respective cultural groupings; (4) *criterion equivalence*, i.e., how the measurement of the variable is interpreted relative to the norms of the respective cultural groupings; (5) *conceptual equivalence*, i.e., the assessment measure is addressing the same theoretical construct in each culture. More recently, Sutrisno et al. (2014) provided an alternative system for categorising forms of equivalence relevant to translation/interpretation: (1) *lexical equivalence* as relating to individual words; (2) *conceptual equivalence* as concerned with ideas or concepts; and (3) *dynamic equivalence* which places a pragmatic emphasis on the message being communicated in a form that is most natural for users of the target language.

To enhance the preparation of assessment measures for transcultural use, van Ommeren et al. (1999) drew on the work of Flaherty et al. (1988) and Manson (1997) to develop a *translation monitoring form*. His approach incorporates lexical back-translation and iterative translation, first by bilingual speakers of the languages involved but also with input from local community members in the target language(s). Emphasis is placed on checking the “comprehensibility, acceptability, relevance and completeness” of the translation (van Ommeren et al., 1999, p. 288). Kohrt et al. (2016) utilised the van Ommeren et al. (1999) approach to produce “a transculturally-translated” (Kohrt et al., 2016, p. 1) version of a depression screening tool (the Patient Health Questionnaire [PHQ-9]) for use in Nepal. This four-stage qualitative process included: (1) forward-translation into Nepalese by bilingual speakers; (2) review by mental health professionals; (3) focus group discussions during which people with a lived experience of distress reviewed each of the items; and (4) blind back-translation by bilingual speakers into the original language. The authors incorporated items that assess local idioms of distress, importantly noting that there is no word for “Depression” in Nepali; instead, the Nepali phrase “*man dukhne*” (which literally translates as “heart-mind pain”) is used to describe a form of emotional distress that the authors proposed was akin to depression. Kohrt et al. (2016) claimed that their qualitative approach optimised “the semantic, technical, content, criterion, and conceptual equivalence of a culturally-adapted tool compared to the original tool” (Kohrt et al., 2016, p. 3).

To maximise the integrity of a translation of the *Bradford Somatic Inventory* (Mumford, 1992), Sumathipala and Murray (2000) employed a panel of nine bilingual (English/Sinhala) students (six of whom were medical students) to develop conceptually and semantically equivalent questions. The process involved individual translation of the instrument by each panel member, and then comparison of these translated versions. If consensus could not be reached, the competing versions of the translations were discussed and the wording for each version was agreed on by the group. These translations were then subjected to ranking leading to the final translation for each question. Sumathipala and Murray (2000) proposed that this approach is preferable to one or two individuals making decisions about the most appropriate translation.

As this summary highlights, there is variation in the standards and approaches used to translate assessment instruments in research studies, and although equivalence of the kinds discussed above is, we would argue, helpful, there is not yet significant attention paid to the risks of epistemic injustice. Efforts to develop greater consensus on the procedures for translating assessment instruments need to be cognisant of the steep epistemic gradient that can exist between the ways in which well-being and distress are conceptualised and communicated in one cultural setting as compared to another, and in one language as compared to another. Extending Bujra's (2006) comment that “translation is more than a technical exercise; it is also a social relationship involving power, status and the imperfect mediation of cultures” (Bujra, 2006, p. 172), such efforts also need to be attentive to how power is exercised in determining what concepts and epistemologies are prioritised in the translation process.

## Interpreted communications about experiences of well-being and distress

Processes of interpretation are a key issue in mental health practice (Swartz et al., 2014), but, to date, the focus has mainly been on technical aspects of interpretation with little attention to the potential for epistemic injustices to arise from possible differences in epistemologies and power dynamics between stakeholders. Temple and Edwards (2002) provides an important counterpoint to this, highlighting a need to acknowledge the “intellectual autobiography” (i.e., the social and epistemic positioning) of different stakeholders in research, especially when involving multiple languages. Other considerations in multi-language research encounters have been highlighted (Chiumento et al., 2017), including interpreter *positionality*, the context of the interpreted communications, and the epistemological underpinnings of these interactions.

During interpretation, an interpreter engages in a process of transferring meaning on the basis of a range of contributory factors including vocabulary, grammar, expression, context, and culture (Esposito, 2001; Regmi et al., 2010). The interpreter is actively involved in the co-construction of knowledge and information; a process that is influenced by their own subjective experiences (Berger, 2015; Temple et al., 2002). Interpreted discussions are not simply dyadic interactions (e.g., interpreter–EBE, and interpreter–health professional) —through the interpreter's involvement, the interactions become triadic (e.g., EBE–interpreter–health professional). Thus, interpreted interactions introduce additional *intersubjectivity* (see Figure 1), an addition which increases the risk of epistemic injustice. Explicit recognition of this intersubjectivity can help promote communications between different stakeholders that are characterised by mutual respect. Given the inherent intersubjectivity of the communication, the process of interpretation can be understood as reconstruction of, rather than discovery of, meaning (Temple & Young, 2004)—a process where a “participant's words are not *recreated* but *re-presented*” (Chiumento et al., 2017, p. 3, emphasis in the original).

Theoretical perspectives from the field of interpretation studies (e.g., Nord, 2014; Williams & Chesterman, 2014) highlighted potential sources of bias that can emerge, and provided advice about proactive steps that can minimise the risks that these biases occur. These include being attentive to source or target language orientation, being mindful of issues of equivalence, and maintaining or altering the register in which communication is spoken. However, whereas professional interpreters may readily practise such procedures, in low-resource settings across the globe, there is a reliance on briefly trained lay-interpreters whose interpreting qualifications and experience may be limited (Chiumento et al., 2017). In post-conflict situations, in order to break down potential barriers related to the involvement of community outsiders (Inhetveen, 2012; Shimpuku & Norr, 2012), it may be more important that the interpreter is situated in the local context rather than being well-qualified in interpretation.

Reflecting on processes of interpretation in research, Temple (2002) note that “interviews are rarely transcribed in the original language, and possible differences in the meanings of words or concepts across languages vanish into the space between spoken otherness and written sameness” (p. 844). Further, in a review of multi-language qualitative research, Squires (2009) highlighted five areas of methodological inconsistency: (1) the translator or interpreter being rendered invisible in the research process; (2) an absence of interview question piloting in the language of participants; (3) a failure to report the training, qualifications, or experience of the translator or interpreter; (4) translation not being explicitly acknowledged as a potential study limitation, and (5) the deployment of methodological frameworks not suited to multi-language research.

Swartz (1996) pointed out that interpreters operate in the context of complex power dynamics that limit their role as linguistic and/or cultural brokers. For example, they may be tempted to interpret material to fit with what they perceive the health professionals expect to hear. Through the quality checks they employed, Williamson et al. (2011) found that interpreters had altered participant responses to concord with perceived expectations of other stakeholders in the communications. We would also contend that it is possible that interpreters may introduce less overt alterations in meaning as interpretation takes place through the intersubjective lens of the stakeholders involved, in turn shaping understanding and communication, as highlighted by Temple (2002). This risk may be increased when interpreters are not sufficiently trained. The use of poorly trained/informal individuals can also present ethical issues in relation to confidentiality and consent (Cross & Bloomer, 2010). Kilian et al. (2014) found that errors made by informal interpreters resulted in patients potentially appearing to be more mentally unwell than they actually were, and that 46% of these errors were likely to have had a negative impact on the goal of the clinical work. As this summary highlights, the intersubjectivities present in interpreted communications bring with them a range of considerations relating to differences in epistemologies and dynamics of power between stakeholders. These issues have important implications for healthcare and research.

## Epistemic brokering

Raymond (2014) introduced the term *epistemic brokering* to capture the role that interpreters can potentially play in “finessing the inherent asymmetries of knowledge in patient–provider encounters, along with interactional contingencies that can arise during the on-going medical encounter” (p. 427). The concept of epistemic brokering, Raymond suggests, provides a framework for understanding the role that interpreters can play as co-diagnostician, gatekeeper, and advocate (Bolden, 2000; Hsieh, 2006; Hsieh & Kramer, 2012). According to Raymond (2014), in addition to linguistic brokering (i.e., facilitating effective communication between speakers of different languages), and cultural brokering (i.e., bridging differences in beliefs and practices between stakeholders from different cultural backgrounds), interpreters (like other stakeholders in the triad) can act as epistemic brokers (discursively (re)shaping knowledge expressed by health professionals for an EBE’s benefit, and vice versa). Epistemic brokering moves beyond surface level differences in language and recognises the social and power implications of the moment-by-moment use of language between the EBE, interpreters, and health professionals. As Raymond notes:

Regardless of the existence or lack of linguistic/cultural equivalents for a given turn-at-talk, interpreters-as-epistemic-brokers take sequential context into account as they work to facilitate the development of common ground between patients and clinicians … not only the transfer of knowledge itself from one interactant to another but also the discursive designs through which such intersubjective understanding is sequentially achieved in talk. (p. 442)

By using transcripts of interpreted consultations between a health professional and EBE, Raymond (2014) identified the risk of health professionals engaging in what he terms *over-supposing* and *under-telling* in their interactions with EBE. For example, the professional might assume that the EBE has existing knowledge of complex terminology and may therefore under-explain concepts. In such situations, an interpreter may choose to broker the epistemic gradient between the stakeholders by presenting material in a less-presupposing way that does not hold the EBE accountable for recognising particular referents in the health professional's questions. According to Raymond, this helps to promote a sense of solidarity between the knowledge position of the EBE and the interpreter and reduces the face costs (Brown & Levinson, 1987) for the EBE in requesting further information about an unfamiliar concept. Equally, epistemic brokering may involve interpreters having to manage health professionals’ assigning a level of knowledge to the EBE that is lower than what they actually have—an under-supposing and over-telling position (Raymond, 2014). When this risk is not mitigated, the EBE may feel patronised. As such, the epistemic brokering of an interpreter can help to ensure that professional knowledge is conveyed in an “interactionally appropriate manner” (Raymond, 2014, p. 433). Importantly, interpreters can also broker health professionals’ access to knowledge held by an EBE, such as when the health professional is in an epistemically downgraded position (Raymond, 2014). Specifically, the interpreter may be able to mediate in situations when an EBE over-supposes and under-tells, or under-supposes and over-tells, in the sharing of their first-hand knowledge and experience. It is important to note here that Raymond's proposal that interpreters can act as epistemic brokers is based on the assumption that the interpreter will be a fair and neutral arbitrator in the process, which may not always be the case.

Incidents of over-supposing/under-telling or under-supposing/over-telling between stakeholders involved in multi-language communications about distress and well-being can contribute to epistemic injustices. To reduce this risk there is a need for: 1) more research exploring epistemic positionality in interpreted multi-language communications about well-being and distress; 2) more explicit emphasis on the need for stakeholder reflexivity in relation to epistemic considerations in interpretation. Equally, epistemic positionality and a lack of epistemic brokering may lead to difficulties emerging in the translation of written materials such as standardised assessment measures that have been shaped by particular epistemic frames. Importantly, there is also a pressing need to raise awareness of the threat that epistemic gradients may have on equitable and mutually purposeful communication about well-being and distress, and to provide approaches aimed at facilitating opportunities for epistemic brokering.

Although resources such as the *Cultural Formulation Interview* (CFI) of the DSM-5 (APA, 2013b) have helped to acknowledge the importance of recognising cultural and linguistic differences in mental health consultations, there is no advice provided on how epistemic gradients identified through the application of such tools might be negotiated. Nor is there guidance on how epistemic gradients should be brokered across different languages. As such, we wish to build on the theory and research that we have discussed in this article to foster opportunities to increase stakeholder reflexivity and critical awareness in multi-language communication about distress and well-being, and highlight how the risk of epistemic injustice in such communication can be mitigated.

## Minimising epistemic injustice in the languaging of well-being and distress

We propose a set of prompts that are intended to promote stakeholder awareness of, and critical reflection about, factors operating at both the macro- and micro-level (as indicated in Figure 1) that influence epistemic divergences and associated communication risks in interpreted or translated multi-language communications. The aim of these prompts is not to act as an objective measure of the accuracy of interpretation. Nor are the prompts intended as a prescription for which epistemic positions should be “accepted” within these communications. Instead, the prompts are intended to facilitate greater parity and reciprocity in multi-language communications relating to well-being and distress by foregrounding the intersubjective space and associated epistemic gradients. As such, these prompts aim to promote inter-epistemic ethics in the interpretation and translation of communication about well-being and distress. The prompts are based on the theoretical perspectives considered in this article, and the *Global Mental Health* field experiences of the authors who have been involved in culturally and linguistically adapting interventions and assessment instruments in Sub-Saharan Africa and South Asia (Andrews et al., 2017, 2018; Burkey et al., 2018).

The prompts can be used to support reflection between stakeholders involved in multi-language communication about distress and well-being. This reflection can involve all stakeholders. However, the prompts can be used to support reflection between the interpreter/translator and at least one other stakeholder (EBE, health professional or researcher—depending on the nature of the communication)—this will allow stakeholders to conduct the discussion in a shared language that they are both fluent in. The reflection should occur immediately (or as soon as possible) after the communication to ensure stakeholders have as complete as possible recollection of the proceedings. We encourage those involved to use the prompts pragmatically rather than rigidly—they are after all intended to facilitate, rather than inhibit, discussion. Therefore, there is scope to explore related themes beyond the prompts where appropriate.

Prompt 1: *Awareness about the concepts and explanatory models employed by people in the communication*. When describing distress and/or well-being, different concepts (or ways of thinking) can be used. Thinking about each person involved in the communication (EBE, researcher/clinician, interpreter/translator), reflect on the breadth of factors (e.g., environmental, physical health-related, psychological, spiritual, religious, social relationships) that were used when explaining and/or describing distress and well-being?

Prompt 2: *Potential differences in how people used concepts and explanatory models employed in the communication.* Think about how each of the people involved in the communication used concepts of distress and/or well-being. Were there differences between the ways in which each person used these concepts? How pronounced were these differences? Please reflect on some relevant examples.

Prompt 3: *Strategies used to develop a shared understanding and how this influenced the nature of the communication*.

- Think back to the recent communication. What strategies were used by each of the people involved to develop a shared understanding of the concepts? For example:
  - Using analogies to illustrate key distress and/or well-being concepts
  - Changing the words used to describe distress and/or well-being to make them easier to understand e.g., from “anxiety” to “worrying a lot”
  - Using illustrations to describe experiences of distress and/or well-being
  - Describing a scenario/experience to illustrate experiences of distress and/or well-being
  - Other (please describe):
- When working together to develop a shared understanding of concepts of distress and/or well-being, some perspectives might change more than others. Thinking back to the communication, how much did each persons’ use of concepts change?
- Whose use of concepts changed the most during the communication (e.g., Expert by Experience, Researcher/Clinician)?

Prompt 4: *The possibility of people “over-supposing/under-telling” in their communication, and how this impacted on the communication.* In communications about distress and/or well-being, it is possible that one or more of the people involved assumes that the other people already know about the concepts being discussed. This kind of assumption is called “over-supposing”. Often, the result is that the person making this assumption does not say enough about the concept or describe/explain it in sufficient detail. This can be called “under-telling”. When over-supposing/under-telling happens, this can make it harder to develop a shared understanding of the concepts.

- Were there examples of the people involved in the interaction *over-supposing/under-telling* during the communication?
- Please provide examples of over-supposing/under-telling that were present in the communication.
- What impact might this over-supposing/under-telling have had on the communication and how people involved in the communication felt?

Prompt 5: *The possibility of people “under-supposing/over-telling” in their communication, and how this impacted on the communication.* In communications about distress and/or well-being, it is possible that one or more of the people involved assumes that the other people did not know about the concepts being discussed. This kind of assumption is called “under-supposing”. Often, the result is that the person making this assumption tells people things that they already know. This can be called “over-telling”. When under-supposing/over-telling happens, this can leave people feeling talked down to, or belittled.

- Were there examples of *under-supposing/over-telling* present in the communication?”
- Please provide examples of *under-supposing/over-telling* that were present in the communication:
- What impact might this *under-supposing and over-telling* have had on the communication and how people involved in the communication felt?

Prompt 6: *Identifying key learning points that can inform future practice.* How might the insights gained through the reflections facilitated by these prompts help shape future similar communications? (e.g., How could an interpreter adjust for over-supposing, etc.?)

The prompts are intended to be illustrative rather than exhaustive. In proposing the prompts, we are aware of the need for us, as authors, to practise the reflexivity that we are encouraging others to engage in. We recognise that the development of these prompts, although influenced by our experiences garnered during more than a decade of multi-language communication in a range of global settings, will have been influenced by our own positionality as Global Mental Health researchers based at UK academic institutions. Notably, being White, English-speaking, European researchers affects our experiences of multi-language encounters. Recognising this, we propose these prompts as a starting point based upon our collective, albeit specific and partial, experiences of multi-language exchanges, and knowledge of relevant literature—including that discussed in this article. We note the importance of an ongoing dialogue between mental health interpreters, researchers, practitioners, and EBE engaged in multi-language communication in a range of clinical and research contexts across the globe to further refine approaches for reflecting on the risk of epistemic injustices. In this way, the prompts can serve as an important first step in methodological and practice innovations aimed at promoting intersubjective reflexivity and reducing the risk of epistemic injustice in multi-language communication about distress and well-being.

## Conclusion

Efforts to understand and address distress and well-being can involve collaboration between stakeholders who bring diverse linguistic and cultural resources to the interactions through which discourses are generated, analysed, interpreted and disseminated. This article has highlighted epistemic and ethical complexities in the way that communication generally, but processes of multi-language translation and interpretation in particular, is handled in research and practice relating to distress and well-being. There is a need for critical interrogation of the way that particular epistemic frames about distress and/or well-being are deployed and can assume hegemonic status in both intersubjective communications, and in processes such as the translation of assessment instruments. Failure to acknowledge and address positionality and power may result in epistemic injustices occurring rather than the equitable sharing of diverse understandings about distress and/or well-being. The prompts proposed in this paper offer a pragmatic approach to facilitating reflexive consideration of how differences in understanding about relevant concepts (e.g., idioms/cultural concepts of distress and/or concepts related to the biopsychosocial model of mental disorders) are handled in multi-language communications about distress and/or well-being. As Summerfield (2012, p. 523) observed, the key issue in multi-cultural mental health research is “not translation between languages, but accurate translation between worlds”.

## Notes

1. The term “epistemic violence” was first introduced by Spivak (1988) in her essay “Can the subaltern speak?”.

2. The Acholi and Langi languages share many similarities but also some important differences (Hammarström, Forkel, Haspelmath, & Bank, 2016).
